# Pulsed-Field Ablation–Induced Coronary Vasospasm for Atrial Tachycardia Originating From the Lateral Tricuspid Annulus

**DOI:** 10.1016/j.jaccas.2025.106153

**Published:** 2025-12-03

**Authors:** Tomoyuki Kouguchi, Yuichiro Sagawa, Kazuya Murata, Hirofumi Arai, Atsuhito Oda, Junichi Kishaba, Yumi Yasui, Yuki Kato, Tetsuo Sasano, Yasuteru Yamauchi

**Affiliations:** a Department of Cardiology, Japan Red Cross Yokohama City Bay Hospital, Kanagawa, Japan; b Department of Cardiovascular Medicine, Institute of Science Tokyo, Tokyo, Japan

**Keywords:** atrial tachycardia, coronary vasospasm, pulsed-field ablation, PulseSelect

## Abstract

**Background:**

Pulsed-field ablation (PFA) is a novel nonthermal technique that achieves myocardial ablation with high tissue selectivity and fewer complications than conventional thermal methods. Coronary vasospasm has been increasingly reported with the Farawave catheter, but data on the PulseSelect system are limited.

**Case Summary:**

A 67-year-old man with paroxysmal atrial fibrillation and atrial tachycardia (AT) underwent catheter ablation. Pulmonary vein isolation was successfully achieved with the PulseSelect system. Subsequent mapping localized the AT to the lateral tricuspid annulus. Radiofrequency ablation repeatedly terminated the AT but was followed by immediate reinduction, failing to provide durable elimination. PulseSelect PFA was then applied to the same site, resulting in immediate AT termination and complete noninducibility thereafter. However, repeat coronary angiography revealed 75% stenosis in the right coronary artery, consistent with vasospasm, which promptly resolved after intracoronary nitroglycerin administration.

**Discussion:**

This case demonstrates that PFA-induced coronary vasospasm can also occur with the PulseSelect system, often without electrocardiographic changes, warranting careful coronary assessment during ablation near atrioventricular annuli.

**Take-Home Message:**

When using the PulseSelect system for ablation of atrioventricular annulus–origin arrhythmias, silent coronary vasospasm can occur regardless of catheter design, necessitating vigilance near the coronary arteries.

## Visual Summary

**Figure:**
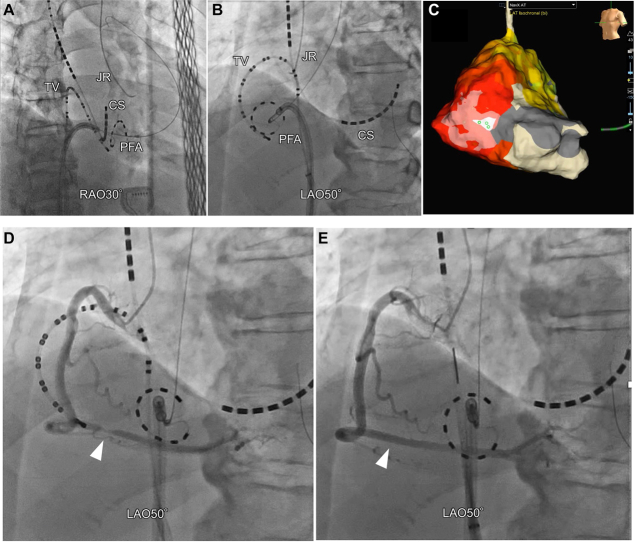

**Visual Summary:**
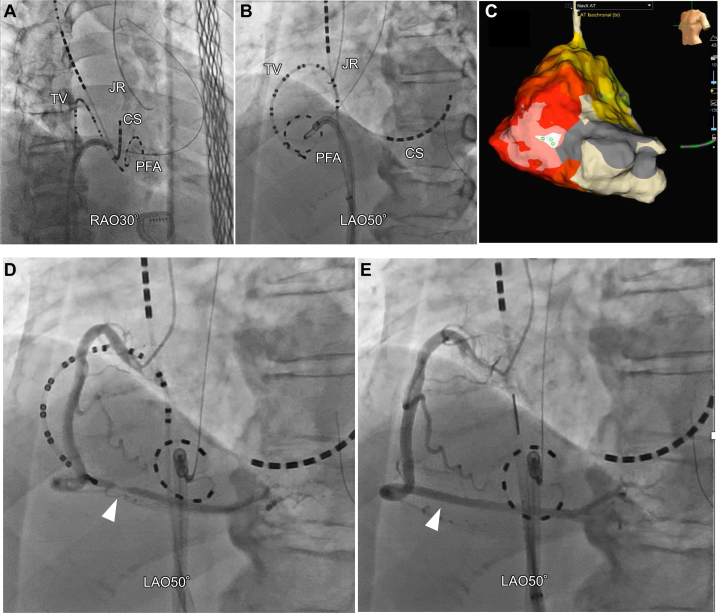
PFA-Induced Coronary Vasospasm for Atrial Tachycardia Originating From the Lateral Tricuspid Annulus A) Angiographic RAO view showing the PulseSelect catheter positioned along the tricuspid annulus. (B) Angiographic LAO view confirming catheter contact with the posterolateral tricuspid annulus. (C) Three-dimensional mapping identified the earliest atrial activation at the 9-o’clock position on the tricuspid annulus. (D) After PFA, coronary angiography demonstrated a 75% stenosis in segment 3 of the right coronary artery. (E) Intracoronary administration of 3 mg nitroglycerin resulted in immediate resolution of the stenosis. CS = coronary sinus catheter; JR = Judkins-right catheter; LAO = left anterior oblique view; PFA = pulsed-field ablation; RAO = right anterior oblique view; TV = tricuspid valve catheter.

Pulsed-field ablation (PFA) is a novel nonthermal ablation technique that enables myocardial tissue ablation with high selectivity, minimizing injury to the adjacent structures such as the esophagus and phrenic nerve. Although PFA is considered relatively safe, recent studies have reported cases of coronary vasospasm, mostly involving the pentaspline Farawave catheter (Boston Scientific). However, limited data are available regarding coronary vasospasm associated with other PFA systems. Here, we report a case of coronary vasospasm induced by a PulseSelect circular-array catheter (Medtronic) during the ablation of atrial tachycardia (AT) originating from the lateral tricuspid annulus. This case emphasizes that vasospasm may occur regardless of catheter design, and it highlights the need for vigilance when ablating near the coronary arteries.

**Take-Home Messages**

• Coronary vasospasm can occur during pulsed-field ablation with the PulseSelect system, not just with the Farawave catheter, demonstrating that this complication is not catheter specific.
• Because vasospasm may develop silently without electrocardiographic changes, coronary angiography or careful coronary assessment should be considered when ablating near the coronary arteries.

## History of presentation

A 67-year-old man was referred to our hospital for evaluation of palpitations and was diagnosed as having symptomatic paroxysmal nonvalvular atrial fibrillation (AF) and atrial tachycardia (AT) by Holter monitoring.

## Past medical history

The patient had a medical history of hypertension. His CHA_2_DS_2_-VASc score was 1. He was prescribed rivaroxaban 15 mg and bisoprolol 2.5 mg, but palpitations recurred.

## Differential diagnosis

The differential diagnoses included AF, AT, atrial flutter, and fast-slow atrioventricular node re-entrant tachycardia.

## Investigations

Transthoracic echocardiography demonstrated a normal left ventricular ejection fraction of 65% and a left atrial diameter of 38 mm. Contrast-enhanced cardiac computed tomography showed no evidence of thrombus in the left atrium or left atrial appendage.

## Management

The patient was diagnosed with paroxysmal AF and AT and was admitted for catheter ablation. Intravenous propofol (4-10 mg/kg/h) was administered for sedation, and mechanical ventilation was managed using an I-gel® supraglottic airway (Intersurgical Ltd). Electroanatomical mapping was performed with the EnSite 3D mapping system (Abbott Medical).

At the start of the procedure, frequent and incessant AT was induced by catheter stimulation. During AT, the V_1_ lead demonstrated a negative P-wave, suggesting a right-sided atrial origin. Because the AT was not sustained, we first performed pulmonary vein (PV) isolation using the PulseSelect circular array catheter. A total of 43 applications were delivered (each consisting of 4 biphasic, bipolar pulses at 1500 V), as follows: left superior PV (8 applications), left inferior PV (12), left PV carina (6), right superior PV (8), and right inferior PV (9). Postablation voltage mapping confirmed successful PV isolation.

**Figure 1:**
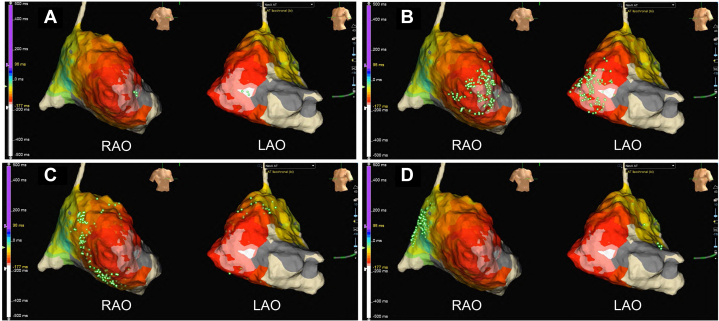
Activation Map During Atrial Tachycardia Panels (A) to (D) depict the sequential propagation of this AT, characterized as a focal AT with the earliest activation observed at the 9 o'clock position on the tricuspid annulus. AT = atrial tachycardia; LAO = left anterior oblique view; RAO = right anterior oblique view.

Subsequently, AT was easily induced, and propagation mapping was performed in the right atrium. Propagation mapping revealed focal AT, with the earliest activation site located at the 9 o'clock position on the tricuspid annulus (Figure 1). Multiple radiofrequency (RF) applications were delivered; however, although AT was repeatedly terminated during ablation, it was easily reinduced with stimulation. This cycle occurred several times, and RF ablation ultimately failed to achieve durable termination of AT. Therefore, the strategy was switched to PFA with the PulseSelect.

**Figure 2:**
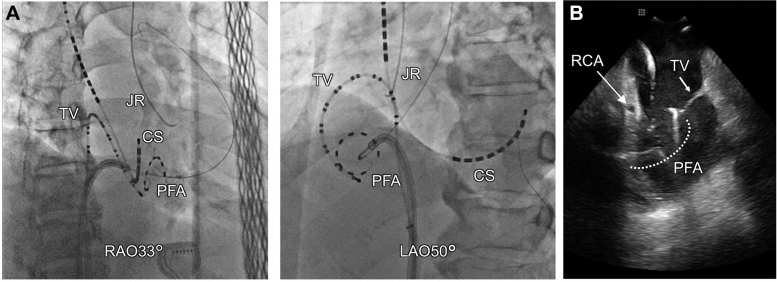
Successful Pulsed-Field Ablation Site (A) Angiographic view of the PulseSelect catheter positioned against the tricuspid annulus in a counterclockwise direction. (B) Intracardiac echocardiography demonstrating stable contact of the PFA catheter with the tricuspid annulus. CS = coronary sinus catheter; JR = Judkins-right catheter; LAO = left anterior oblique view; PFA = pulsed-field ablation; RAO = right anterior oblique view; RCA = right coronary artery; TV = tricuspid valve catheter.

**Figure 3:**
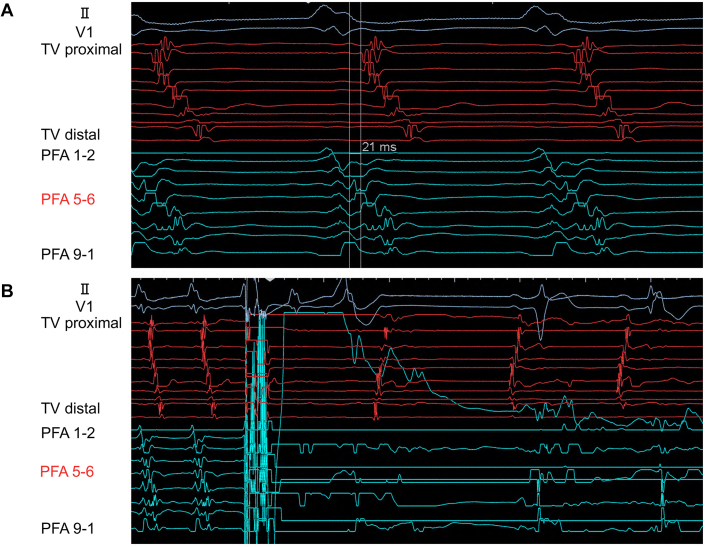
Intracardiac Electrograms During Atrial Tachycardia and Pulsed-Field Ablation (A) Local atrial electrogram at PFA 5-6 represented the earliest atrial activation site, preceding the onset of the P-wave by 21 ms during AT. (B) A single PFA application successfully terminated the AT. AT = atrial tachycardia; PFA = pulsed-field ablation.

Prior to PFA, coronary angiography (CAG) confirmed the absence of any significant coronary artery stenosis. The PulseSelect catheter guidewire was advanced into the right ventricular outflow tract, and the PulseSelect catheter was positioned counterclockwise along the tricuspid annulus. Intracardiac echocardiography (ICE) confirmed stable catheter-tissue contact, demonstrating that the right coronary artery was in close proximity to the tricuspid annulus (Figure 2). Local atrial electrograms (PFA 5-6) preceded the onset of the surface P-wave by 21 ms during AT. A single PFA application resulted in the termination of AT (Figure 3), after which an additional PFA application was delivered.

**Figure 4:**
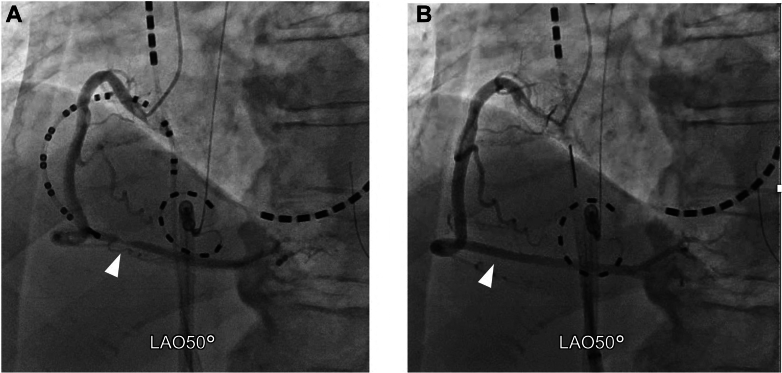
Right Coronary Angiogram Immediately After Pulsed-Field Ablation and Following Intracoronary Nitroglycerin Injection (A) After PFA applications, CAG revealed 75% stenosis in segment 3 of the right coronary artery (arrowhead). (During CAG, the PFA catheter was floated in the center of the tricuspid valves.) (B) A total of 3 mg of intracoronary nitroglycerin was administered, resulting in immediate resolution of the stenosis (arrowhead). CAG = coronary angiography; PFA = pulsed-field ablation; RCA = right coronary artery.

Although no electrocardiographic changes were observed, repeat CAG revealed 75% stenosis in segment 3 of the right coronary artery (Figure 4A), suspected to represent coronary vasospasm. Two intracoronary injections of nitroglycerin (1.5 mg each) were administered, leading to immediate resolution of the stenosis (Figure 4B). Finally, we attempted to reinduce AT using atrial stimulation and found it could no longer be induced.

## Outcome and follow-up

The patient remained free of both AF and AT throughout the 6-month follow-up period.

## Discussion

In the current case, AT originating from the tricuspid annulus could not be terminated with RF energy; however, a single application of the PulseSelect PFA catheter successfully eliminated arrhythmia. One possible explanation for the failure of RF ablation was the difficulty in achieving stable catheter-tissue contact at the tricuspid annulus. By anchoring the PulseSelect catheter guidewire in the right ventricular outflow tract, we were able to improve catheter stability and ensure adequate contact, thereby enabling successful ablation.

PFA is characterized by tissue selectivity and has been associated with fewer complications than conventional thermal ablation, including pulmonary vein stenosis, esophageal injury, and phrenic nerve damage.^1^^,^^2^ Nevertheless, coronary vasospasm is an increasingly recognized but rare PFA-related complication.^3^ Most published cases have involved the Farawave catheter, whereas reports describing vasospasm associated with the PulseSelect system are limited. Although the pentaspline Farawave and the circular-array PulseSelect differ in electric field strength and design,^4^ in the present case vasospasm occurred with the PulseSelect catheter, indicating that this complication is not exclusive to a particular catheter design but may represent an intrinsic risk of PFA.

Although prophylactic nitroglycerin administration before cavotricuspid isthmus ablation has been reported to reduce coronary vasospasm,^5^ we elected not to administer it given the scarcity of vasospasm reports with PulseSelect catheters. This case nevertheless demonstrates that vasospasm can occur with this system, particularly when ablation is performed near the coronary arteries. ICE confirmed the close proximity of the right coronary artery to the tricuspid annulus, thereby highlighting the utility of ICE in anticipating vasospasm risk and guiding consideration of prophylactic vasodilator use.

Most European reports indicate that approximately 90% of PFA-related coronary vasospasms are accompanied by electrocardiographic changes.^6^ Interestingly, no such changes were observed in our patient; we speculate that this was because of the presence of only moderate (approximately 75%) coronary artery stenosis, without major hemodynamic compromise. This finding indicates that vasospasm can occur silently, without electrocardiographic evidence. Therefore, CAG should be considered after PFA when ablation targets are located near the coronary arteries, even in the absence of electrocardiographic changes.

This case further suggests that the incidence of PFA-induced coronary vasospasm may be underestimated, as routine angiographic evaluation is not commonly performed during ablation procedures. Silent vasospasm, such as in the present case, may be more frequent than currently appreciated, and it could remain undetected without direct imaging. Although vasospasms are generally transient and reversible, recurrent or subclinical episodes may contribute to microvascular dysfunction or predispose patients to ischemic complications over time.

The mechanisms underlying PFA-induced vasospasm remain incompletely understood. Potential explanations include direct electroporation of vascular smooth muscle, autonomic reflex activation, and transient endothelial dysfunction. Elucidating these mechanisms may inform preventive strategies, including prophylactic vasodilator administration, optimization of catheter positioning, and adjustments to pulse delivery protocols.

Taken together, the present findings demonstrate that coronary vasospasm is a potential complication across different PFA catheter designs. As PFA is increasingly applied beyond pulmonary vein isolation to more anatomically challenging substrates, procedural planning should incorporate careful assessment of coronary anatomy, attention to catheter positioning, and consideration of prophylactic vasodilator therapy when ablation is performed in close proximity to the coronary arteries.

Although acute coronary spasm can be mitigated by prophylactic nitroglycerin, PFA performed near the coronary arteries has recently been reported to cause chronic intimal thickening. Tam et al^7^ reported that patients undergoing PFA at the cavotricuspid or mitral isthmus exhibited an approximately 10% reduction in luminal diameter on repeat CAG 3 months later, highlighting the importance of long-term surveillance, including coronary computed tomography angiography. Although significant high-grade stenosis has not been observed, mild coronary narrowing may occur. Therefore, when PFA is performed near the atrioventricular annulus, preprocedural CAG should be considered to exclude pre-existing organic stenosis. In addition, when ablation is performed at the atrioventricular annulus, careful follow-up is warranted to monitor for potential progression of coronary stenosis during the chronic phase.

## Conclusions

This case demonstrates that coronary vasospasm can occur even with the PulseSelect PFA system during ablation near the tricuspid annulus. Although no electrocardiographic changes were observed, CAG revealed significant vasospasm, underscoring the importance of coronary assessment even in asymptomatic cases. Heightened awareness of this potential complication is essential when performing PFA in proximity to the coronary arteries.

## Funding Support and Author Disclosures

The authors have reported that they have no relationships relevant to the contents of this paper to disclose.
